# Supplementary material for: Effect of Exercise Duration on Postprandial Glycaemic and Insulinaemic Responses in Adolescents
Source: Nutrients. 2020 Mar 12;12(3):754. doi: 10.3390/nu12030754 (PMC7146363; doi:10.3390/nu12030754)
Supplement: Supplementary file 1 [file nutrients-12-00754-s001.pdf]

Supplementary Table 1: Blood glucose concentration [mmol·L^{-1}] and plasma insulin concentration [pmol·L^{-1}] across day 1 and day 2 of the 30 min LIST, 60 min LIST and resting control trials, for all participants and split by girls and boys

		Day 1						Day 2			
		Fasted	Post-exercise	60 min post-exercise	30 min post-lunch	60 min post-lunch	120 min post-lunch	Fasted	30 min post-breakfast	60 min post-breakfast	120 min post-breakfast
Blood Glucose Concentration [mmol·L^{-1}]											
30 min LIST trial	Combined	4.0 ± 0.6	4.6 ± 0.7	3.6 ± 0.6	5.1 ± 0.8	4.3 ± 0.5	4.4 ± 0.6	3.9 ± 0.6	5.7 ± 1.0	4.4 ± 0.9	4.4 ± 0.7
	Girls	3.8 ± 0.7	4.4 ± 0.5	3.6 ± 0.6	5.0 ± 0.9	4.2 ± 0.6	4.4 ± 0.5	3.9 ± 0.6	5.6 ± 1.0	4.2 ± 0.9	4.4 ± 0.6
	Boys	4.1 ± 0.6	4.8 ± 0.9	3.7 ± 0.6	5.2 ±0.5	4.5 ± 0.4	4.6 ± 0.7	4.0 ±0.6	5.8 ± 1.0	4.6 ±0.8	4.3 ± 0.8
60 min LIST trial	Combined	3.9 ± 0.6	4.6 ± 0.7	3.8 ± 0.5	5.1 ± 1.0	4.4 ± 0.8	4.3 ± 0.8	4.0 ± 0.7	5.8 ± 1.0	4.4 ± 0.8	4.3 ± 0.8
	Girls	3.8 ± 0.7	4.5 ± 0.7	3.8 ± 0.6	5.0 ± 1.0	4.3 ± 0.8	4.1 ± 0.7	3.8 ± 0.6	5.8 ± 1.1	4.3 ± 0.7	4.2 ± 0.9
	Boys	4.1 ± 0.4	4.8 ± 0.7	3.8 ± 0.5	5.4 ± 1.0	4.5 ± 0.8	4.6 ± 0.8	4.3 ±0.7	5.9 ±0.7	4.5 ± 0.8	4.4 ±0.7
Resting control trial	Combined	4.2 ± 0.8	4.4 ± 09	4.1 ± 0.9	5.0 ± 0.9	4.3 ± 0.6	4.2 ± 0.7	3.9 ± 0.7	5.8 ± 1.1	4.5 ± 0.9	4.4 ± 0.6
	Girls	4.2 ± 0.8	4.1 ± 0.8	4.0 ± 0.7	4.9 ± 1.0	4.2 ± 0.6	4.1 ± 0.6	3.7 ± 0.7	5.8 ± 1.3	4.4 ± 1.0	4.3 ± 0.6
	Boys	4.2 ± 0.8	4.8 ± 0.8	4.2 ±1.0	5.2 ± 0.7	4.4 ± 0.6	4.5 ±0.8	4.0 ± 0.6	5.8 ± 0.8	4.6 ± 0.8	4.5 ±0.6
Plasma Insulin Concentration [pmol·L^{-1}]											
30 min LIST trial	Combined	61.3 ± 24.1	157.3 ± 72.6	85.2 ± 75.0	323.2 ± 145.6	204.8 ± 113.6	154.5 ± 69.0	68.5 ± 49.9	449.1 ± 197.2	242.9 ± 101.3	213.3 ± 122.2
	Girls	66.3 ± 23.3	169.5 ± 80.4	94.4 ± 61.2	374.3 ± 153.2	235.6 ± 125.4	177.5 ±70.4	64.9 ± 35.2	513.5 ± 206.2	251.4 ± 108.3	25.4 ± 125.5
	Boys	54.1 ± 24.0	139.6 ± 57.5	72.6 ± 91.3	249.8 ± 97.8	160.4 ± 78.1	120.7 ± 52.4	73.7 ± 66.7	356.5 ± 143.7	229.8 ± 91.7	152.7 ± 89.8
60 min LIST trial	Combined	59.9 ± 40.9	158.3 ± 75.6	58.3 ± 46.7	271.0 ± 151.4	195.6 ± 130.8	161.4 ± 103.0	60.1 ± 56.8	451.0 ± 205.0	263.2 ± 127.3	165.5 ± 108.2
	Girls	67.8 ± 48.8	153.6 ± 75.6	58.8 ± 45.8	309.0 ± 168.9	223.9 ± 151.7	181.4 ± 110.0	56.0 ± 42.8	506.4 ± 217.3	279.3 ± 150.1	191.4 ± 111.4
	Boys	47.9 ± 20.4	165.4 ± 120.8	57.6 ± 49.6	216.3 ± 104.0	155.0 ± 81.2	132.7 ± 87.5	66.4 ± 74.7	371.3 ± 160.6	239.9 ± 84.0	128.2 ± 94.5
Resting control trial	Combined	71.1 ± 64.4	132.7 ± 77.8	89.3 ± 82.2	344.7 ± 198.6	251.3 ± 173.3	168.2 ± 103.6	65.3 ± 55.7	496.6 ± 245.1	282.5 ± 164.4	187.2 ± 173.2
	Girls	71.6 ± 72.0	150.3 ± 77.5	84.7 ±57.4	411.8 ± 216.0	278.8 ± 201.2	182.5 ± 113.7	65.9 ± 36.9	572.8 ± 250.7	336.0 ±183.9	222.9 ± 186.0
	Boys	70.2 ± 53.0	107.5 ± 73.3	95.4 ± 108.5	248.4 ± 121.0	211.9 ± 117.9	147.7 ± 86.4	64.4 ± 77.8	387.0 ± 195.6	205.5 ± 90.2	135.8 ± 143.4
